# Prediction of Perioperative Cardiac Events through Preoperative NT-pro-BNP and cTnI after Emergent Non-Cardiac Surgery in Elderly Patients

**DOI:** 10.1371/journal.pone.0121306

**Published:** 2015-03-23

**Authors:** Jinling Ma, Qian Xin, Xiujie Wang, Meng Gao, Yutang Wang, Jie Liu

**Affiliations:** 1 Department of Geriatric Cardiology, Chinese PLA General Hospital, Beijing, China; 2 Department of Cardiology, Chinese PLA General Hospital, Beijing, China; 3 Department of Radiology, Zhaoyuan People's Hospital, Shandong, China; 4 Emergency Department, Chinese PLA General Hospital, Beijing, China; Harvard Medical School, UNITED STATES

## Abstract

**Objectives:**

Clinical risk stratification has an important function in preoperative evaluation of patients at risk for cardiac events prior to non-cardiac surgery. The aim of this study was to determine whether the combined measurement of pre-operative N-terminal pro-brain natriuretic peptide (NT-pro-BNP) and cardiac troponin I (cTnI) could provide useful prognostic information about postoperative major adverse cardiac events (MACE) within 30 days in patients aged over 60 years undergoing emergent non-cardiac surgery.

**Methods:**

The study group comprised 2519 patients aged over 60 years that were undergoing emergent non-cardiac surgery between December 2007 and December 2013. NT-pro-BNP and cTnI were measured during hospital admission. The patients were monitored for MACE (cardiac death, non-fatal myocardial infarction, or cardiac arrest) during the 30-day postoperative follow-up period.

**Results:**

MACE occurred in 251 patients (10.0%). Preoperative NT-pro-BNP and cTNI level were significantly higher in the individuals that experienced MACE than in those who did not (P < 0.001). The confounding factors of age, sex, co-morbidities and preoperative medications were adjusted in a multivariate logistic regression analysis. This analysis showed that preoperative NT-proBNP level > 917 pg/mL (OR 4.81, 95% CI 3.446–6.722, P < 0.001) and cTnI ≥ 0.07 ng/mL (OR 8.74, 95% CI 5.881–12.987, P < 0.001) remained significantly and independently associated with MACE after the adjustment of the confounding factors. Kaplan-Meier event-free survival curves demonstrated that patients with preoperative simultaneous NT-proBNP level > 917 pg/mL and cTnT ≥0.07 ng/mL had worse event-free survival than individual assessments of either biomarker.

**Conclusion:**

Preoperative plasma NT-proBNP and cTnI are both independently associated with an increased risk of MACE in elderly patients after emergent non-cardiac surgery. The combination of these biomarkers provides better prognostic information than using either biomarker separately.

## Introduction

Clinical risk stratification has an important function in preoperative evaluation of patients at risk for cardiac events prior to non-cardiac surgery [1]. However, emergency surgery patients often have limited preoperative physical activity that can provide accurate assessment of cardiac risk [2]. Multiple studies have demonstrated that preoperative N-terminal pro-brain natriuretic peptide (NT-pro-BNP) is a valuable predictor of perioperative cardiovascular complications after non-cardiac surgery [2–13]. The guidelines of the European Society of Cardiology and the European Society of Anesthesiology for preoperative cardiac risk assessment have recommended the consideration of preoperative NT-pro-BNP measurement in high-risk non-cardiac surgery patients [14]. Moreover, accumulating evidence support that troponins reflect minor myocardial injury, thereby providing prognostic information [15,16]. The availability of powerful cardiovascular biomarkers, such as troponins and NT-pro-BNP, offer the opportunity for further refinement of clinical scores [17].

In the present study, we evaluated the value of preoperative NT-pro-BNP and cardiac troponin I (cTnI) levels in a cohort of patients undergoing emergent non-cardiac surgery. Ageing patients have increased risk of perioperative cardiac events because of multiple comorbidities [12]. This prospective observational study aimed to determine whether or not the combined measurement of pre-operative NT-pro-BNP and cTnI can provide useful prognostic information about postoperative major adverse cardiac events (MACE) within 30 days in patients aged over 60 years undergoing emergent non-cardiac surgery.

## Methods

### Study population

Consecutive patients aged 60 years and over presenting for emergent non-cardiac surgery (defined as surgery that must be performed within 24 h after admission) under general anesthesia between December 2007 and December 2013 in Chinese PLA General Hospital and Zhaoyuan People's Hospital were prospectively included in the study. Prior to surgery, patient characteristics, as well as medical and demographic details were documented. These emergent non-cardiac surgeries include abdominal, gynecological, urological, reconstructive, orthopedic, and vascular surgeries. We excluded patients who had severe degree of valvular heart disease and those who were receiving hemodialysis or peritoneal dialysis for renal failure. Patients who are unable to provide informed consent were also excluded. A total of 2519 patients were enrolled in this study.

The written informed consents were obtained from all subjects or their designated relatives. The study was approved by the Ethics Committee of the Chinese PLA General Hospital (Beijing, China) and Zhaoyuan People's Hospital (Shandong, China).

### Measurement of plasma NT-pro-BNP and cTnI

Peripheral blood samples for NT-pro-BNP and cTnI were obtained upon admission at the hospital through direct vein puncture. NT-proBNP and cTnI were measured by electrochemiluminescence immunoassay on the Dimension Vista 500 Intelligent Laboratory System (Siemens Healthcare Diagnostics, Deerfield, Illinois, United States). An increased level of cTnI was defined as ≥ 0.07 ng/mL.

### Follow-up

After surgery, the patients were followed up for 30 days by a research assistant. No patients were lost during follow-up. The patients were monitored for MACE, namely, cardiac death, non-fatal myocardial infarction (MI), or cardiac arrest, during the perioperative period. Non-fatal MI was defined according to the new universal definition of MI. This definition was the typical increase/decrease of troponin together with the evidence of myocardial ischemia with at least one of the following: symptoms of ischemia, ECG changes indicative of new ischemia or new Q waves; or imaging evidence of new regional wall motion abnormality [18]. Cardiac death was defined as death secondary to MI, arrhythmia, or heart failure. Cardiac arrest was defined as a cardiopulmonary event that led to the initiation of cardiopulmonary resuscitation and advanced cardiac life support protocols.

### Statistical analysis

Normally distributed continuous data were expressed as mean ± SD and compared using Student’s t test. Non-normally distributed continuous data were expressed as median with the inter-quartile range and compared using the Mann Whitney U test. The categorical variables were presented as proportions (percentages). Categorical variables were compared with χ^2^ tests. Logistic regression analysis was used to predict the prevalence of MACE, with adjustments for age, sex, co-morbidities, history, and medication. The results are presented as adjusted odds ratios (OR) and their 95% confidence interval (CI). A Kaplan-Meier analysis was performed to assess event-free survival. The event-time curve was separated into four curves according to the discriminatory preoperative NT-pro-BNP and cTnI and these curves were compared by log-rank test. All data were processed using the PASW (version 18.0; SPSS, Chicago, IL, USA). A *P* value <0.05 was considered statistically significant.

## Results

A total of 2519 consecutive patients undergoing emergency surgery between December 2009 and December 2013 were included in this study. The mean age of the subjects was 77.3 ± 8.4 years, and men comprised 52.1% of the group. Of the patients, 1078 (42.8%) underwent abdominal surgery, 807 (32.0%) orthopedic surgery, 212 (8.4%) urological surgery, and 422 (16.8%) other surgery, respectively.

**Fig 1:**
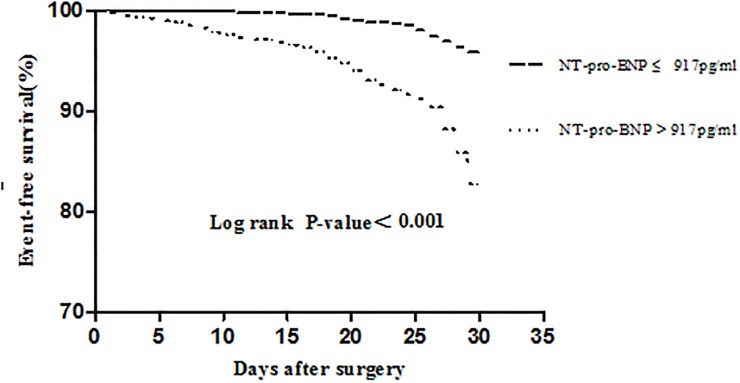
Kaplan-Meier event-time curve according to preoperative NT-pro-BNP levels.

During the 30-day postoperative follow-up period, a total of 251 (10.0%) patients experienced MACE, including 223 nonfatal myocardial infarctions, 11 nonfatal cardiac arrests, and 17 cardiac deaths. The preoperative baseline characteristics of the study population according to the occurrence of MACE during follow-up are presented in Table 1. The preoperative NT-pro-BNP and cTNI level were significantly higher in the individuals that experienced MACE than in those who did not (*P* < 0.001). Moreover, age and chronic renal insufficiency were significantly higher in patients with perioperative MACE than in those without (Table 1). No significant difference was observed in the proportion of subjects with preoperative medications. According to the preoperative NT-pro-BNP, the population was divided into two groups: NT-pro-BNP > 917 pg/mL (n = 1131, 8.3%) and ≤ 917 pg/mL (n = 1388, 91.7%) (Fig. 1).

**Table 1:** Clinical characteristics according to the occurrence of postoperative 30-day MACE.

Characteristic	MACE (+)	MACE (−)	*p* Value
	n = 251	n = 2268	
Age, years	82.4 ± 8.6	76.8 ± 8.2	<0.001
Male, n (%)	141 (56.2)	1171 (51.6)	0.1715
Co-morbidities, n (%)			
Hypertention	177 (70.5)	1566 (69.0)	0.6231
Hypertipidaemia	97 (38.6)	845 (37.3)	0.6663
Diabetes	75 (29.9)	665 (29.3)	0.8535
Atrial fibrillation	33 (13.1)	246 (10.8)	0.2704
COPD	40 (15.9)	346 (15.3)	0.7764
Malignancy	25 (9.9)	221 (9.7)	0.9129
Chronic renal insufficiency	42 (16.7)	116 (5.1)	<0.001
History, n (%)			
Previous myocardial infarction	25 (9.9)	189 (8.3)	0.3804
Previous congestive heart failure	23 (9.2)	189 (8.3)	0.6531
Preoperative NT-pro-BNP (pg/mL)	5198.0 (256.0–12567.0)	803.0 (102.0–2109.0.)	<0.001
Preoperative cTNI (ng/mL)	0.06 (0.00–0.60)	0.03 (0.00–0.1)	<0.001
Preoperative medications, n (%)			
Beta-blockers	109 (43.4)	972 (42.8)	0.8628
Statins	133 (52.9)	1125 (49.6)	0.3088
Calcium channel blocker	57 (22.7)	558 (24.6)	0.5075
Diurets	81 (32.3)	645 (28.4)	0.2035
ACEI/ARB	102 (40.6)	952 (41.9)	0.6835

COPD: chronic obstructive pulmonary disease; ACE: angiotensin converting enzyme inhibitor; ARB: angiotensin receptor blocker.

The confounding factors of age, sex, co-morbidities, and preoperative medications were adjusted in a multivariate logistic regression analysis. This analysis showed that preoperative NT-pro-BNP > 917 pg/mL (OR 4.81, 95% CI 3.446–6.722, *P* < 0.001) and cTNI ≥ 0.07 ng/mL (OR 8.74, 95% CI 5.881–12.987, *P* < 0.001) remained significantly and independently associated with MACE (Table 2).

**Table 2:** Risk factors associated with MACE in a multivariable logistic regression analysis.

Risk factors	*P* Value	OR	95% CI
Age, years	<0.001	1.08	1.006–1.096
Male (%)	0.1587	1.24	0.920–1.666
Co-morbidities, n (%)			
Hypertention	0.8116	0.94	0.585–1.523
Hypertipidaemia	0.1762	1.37	0.867–2.177
Diabetes	0.3010	0.81	0.543–1.208
Atrial fibrillation	0.1953	1.37	0.850–2.210
COPD	0.4847	1.15	0.773–1.719
Malignancy	0.9665	1.01	0.619–1.650
Chronic renal insufficiency	<0.001	3.95	2.526–6.185
History, n (%)			
Previous myocardial infarction	0.5089	1.20	0.695–2.081
Previous congestive heart failure	0.6578	0.88	0.497–1.554
Preoperative NT-pro-BNP>917 pg/mL	<0.001	4.81	3.446–6.722
Preoperative cTNI ≥ 0.07 ng/mL	<0.001	8.74	5.881–12.987
Preoperative medications, n (%)			
Beta-blockers	0.8714	1.03	0.749–1.406
Statins	0.4513	1.14	0.808–1.614
Calcium channel blocker	0.1407	0.75	0.517–1.098
Diurets	0.4283	1.16	0.799–1.696
ACEI/ARB	0.6061	0.91	0.642–1.295

COPD: chronic obstructive pulmonary disease; ACE: angiotensin converting enzyme inhibitor; ARB: angiotensin receptor blocker.

Kaplan-Meier event-free survival curves demonstrated that patients with preoperative NT-pro-BNP level > 917 pg/mL had worse event-free survival compared with those with NT-pro-BNP levels ≤ 917 pg/mL during the 30-day postoperative follow-up period (Fig. 1).

**Fig 2:**
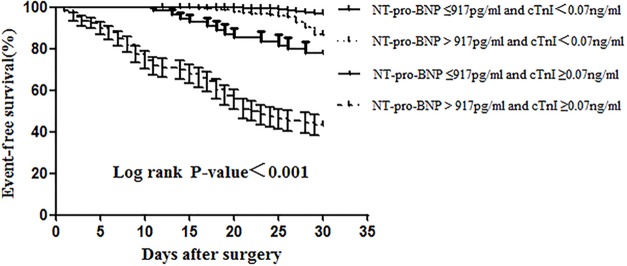
Kaplan-Meier survival curves according to preoperative NT-pro-BNP and cTnI levels

To determine the potential utility of simultaneous cTnI and NT-pro-BNP assessment, the patients were divided into four groups based on cTnT and NT-pro-BNP cut-off points. Patients with elevated levels of both cTnI and NT-pro-BNP had a significantly increased risk (*P* < 0.001) (Fig. 2). Thus, the assessment of both cTnT and NT-proBNP was more effective at identifying a high-risk subgroup than individual assessments of either biomarker.

## Discussion

It is very important to determine the risk of cardiac complications before emergent non-cardiac surgery in elderly patients who already possibly had heart disease, various comorbidities, and whose response to transfusion or fluid therapy is weakened. Furthermore, an emergency population does not have the luxury of intense preoperative cardiovascular workup compared with an elective population because delay to surgery can be detrimental to the outcome [5].

This study provides a comprehensive analysis of the prognostic value of preoperative NT-pro-BNP, alone or in combination with cTnI, in a cohort of patients undergoing emergent non-cardiac surgery. This study demonstrated that preoperative NT-pro-BNP levels are associated with increased risk of MACE during the 30-day postoperative follow-up period in the enrolled elderly patients. Perioperative cardiovascular risks may increase as NT-pro-BNP concentrations increase. After multivariate adjustment for age, sex, co-morbidities, and preoperative medications, logistic regression analysis showed that both pre-operative NT-pro-BNP and cTnI levels are independent predictors of adverse cardiac events. This study demonstrated that a single pre-operative measurement of NT-pro-BNP provides useful information for use in risk stratification. Furthermore, the combined use of NT-pro-BNP and cTnI provided incremental prognostic information that enhances the prediction of increased risk of perioperative MACE. Therefore, a simultaneous increase in both markers identifies patients with high probability of postoperative cardiac events. Simultaneous assessment of the two biomarkers would be more useful in predicting postoperative cardiac events than using either biomarker separately.

In agreement with our results, previously published studies have suggested that an independent association exists between elevated preoperative NT-pro-BNP levels and increased risks of adverse perioperative cardiovascular outcomes [2–5, 19–26]. Measuring NT-pro-BNP in adults having major non-cardiac surgery significantly improves the preoperative risk stratification and can easily be incorporated into clinical practice. This procedure allows physicians to plan prophylactic strategies in patients identified as high risk [27]. The results from these investigations suggest that NT-pro-BNP is useful in predicting perioperative adverse outcome. In the present study, NT-pro-BNP remained an independent correlate of perioperative cardiac events, even when cTnI was included in the multivariable analysis. Previous studies have used different thresholds, from 201 pg/mL to 3980 pg/mL for preoperative NT-pro-BNP assays, to represent abnormal values [7,11,12,20–22]. Although there is no consensus of what the normal NT-pro-BNP values are, we identified 917pg/mL as the optimal cut-off value to predict the 30-day MACE according to another study.^9^ The cutoff point with the best specificity and sensitivity in relation to the primary outcome was 917 pg/mL for preoperative NT-pro-BNP [9].

During the perioperative period, the pathophysiology of perioperative myocardial infarction has been explained by responses to perioperative surgical stress represented by a catecholamine surge with associated hemodynamic stress, systemic inflammation, and hypercoagulability [28,29]. Moreover, a number of other stressors that can lead to myocardial dysfunction exist, such as hypoxia, activation of the sympathetic system, and an increase in plasma pro- and anti-inflammatory cytokines [30]. These factors can increase the risk for postoperative complications.

Previous studies have found that troponin increase occur silently and is a prognostic marker of cardiovascular complications and death after non-cardiac surgery [31–34]. cTnI is unique to myocardium, cTnT can be re-expressed in skeletal muscle in response to injury [35]. An increase in specific cardiac biomarkers, as it is the case for natriuretic peptides and troponins, *always* indicates that the heart is under a stress condition or even actually injured [36]. Our data further confirmed a significant association between cTnI and post-operative cardiac events that is incremental to NT-pro-BNP. The combination of both biomarkers is associated with a substantially higher risk compared with either biomarker alone. The combination of both biomarkers may further improve the identification of patients with increased myocardial wall tension and minor myocardial damage, even in the absence of ischemic symptoms. Our study suggests that the combined use of preoperative NT-pro-BNP and cTnI may be a superior short-term prognostic marker after emergent non-cardiac surgery in elderly patients. Moreover, to improve perioperative risk stratification in noncardiac surgery and going beyond established clinical scores with the use of cardiac biomarkers, prospective studies are needed, specifically aimed at evaluating the independence of troponin and NP predictive value even in mid- and long-term periods [37].

This study has several limitations that should be considered. First, the patient mean age was 77.3 ± 8.4 years in our patient cohort. Therefore, these characteristics may limit the generalizability of our findings to those ages. Second, long-term follow-up after discharge was not performed. The study did not prioritize the long-term follow-up because most postoperative cardiovascular events develop in the early postoperative periods [38, 39]. Third, clearance of NT-pro-BNP is largely dependent on excretion from the kidney [40]. NT-pro-BNP level and its prognostic ability can be affected by renal failure [26, 41]. In this study, we did not exclude patients with renal dysfunction, which may have decreased the specificity of NT-pro-BNP in predicting cardiac complications and mortality. In addition, in the present study, NT-proBNP was measured by electrochemiluminescence immunoassay, which is thought to measure only nonglycoNT-proBNP. Glycosylated proBNP is a major molecular form in human plasma and glycosylated NT-proBNP is underestimated by the NT-proBNP assay system currently being used [40]. Under these conditions, correct interpretation of the NT-proBNP levels and clinical application may require careful consideration. Fourth, a consensus concerning the reference range of perioperative NT-pro-BNP values has not been achieved. We identified 917 pg/mL as the optimal cut-off value. This value was in accordance to the basis of our experience and another study [9]. The optimal discriminatory (or ‘cut-off’) point requires further investigation. Fifth, differences in pre-operative risk factors other than those included in the multivariate model may have affected the MACE results, such as the type of surgery, scope of operation and scores of serious illness. Thus, we should interpret our results very carefully. Finally, this study was performed at two different hospitals. Most of the patients were from North China; thus, our findings may not be completely representative of the population of other locations. Further prospectively multiple center studies with a large number of patients in various surgery are required to form stronger conclusions. Although caution is necessary for the interpretation of our data, we consider it improbable that these limitations have influenced our main findings.

In conclusion, the present study demonstrated that elevated levels of preoperative plasma NT-pro-BNP and cTnI are both independently associated with an increased risk of MACE in a real-life cohort of elderly patients undergoing emergent non-cardiac surgery. Thus, the combination of preoperative NT-pro-BNP and cTnI measurements provide better prognostic information than using either biomarker separately. Patients at high risk should be considered for less invasive procedures and must receive optimized perioperative care. Further research with a larger number of patients having various types of surgeries is needed to confirm the clinical utility of these prognostic tests.
